# Genomics of Aerobic Photoheterotrophs in Wheat Phyllosphere Reveals Divergent Evolutionary Patterns of Photosynthetic Genes in *Methylobacterium* spp.

**DOI:** 10.1093/gbe/evz204

**Published:** 2019-09-17

**Authors:** Athanasios Zervas, Yonghui Zeng, Anne Mette Madsen, Lars H Hansen

**Affiliations:** 1 Section of Environmental Microbiology and Biotechnology, Department of Environmental Science, Aarhus University, Roskilde, Denmark; 2 Aarhus Institute of Advanced Studies, Aarhus University, Denmark; 3 National Research Centre for the Working Environment, Copenhagen, Denmark; 4 Environmental Microbial Genomics Group, Department of Plant and Environmental Sciences, University of Copenhagen, Frederiksberg, Denmark

**Keywords:** AAP bacteria, bacteriochlorophyll, phyllosphere, MALDI-TOF MS, Nanopore, photosystems

## Abstract

Phyllosphere is a habitat to a variety of viruses, bacteria, fungi, and other microorganisms, which play a fundamental role in maintaining the health of plants and mediating the interaction between plants and ambient environments. A recent addition to this catalogue of microbial diversity was the aerobic anoxygenic phototrophs (AAPs), a group of widespread bacteria that absorb light through bacteriochlorophyll α (BChl *a*) to produce energy without fixing carbon or producing molecular oxygen. However, culture representatives of AAPs from phyllosphere and their genome information are lacking, limiting our capability to assess their potential ecological roles in this unique niche. In this study, we investigated the presence of AAPs in the phyllosphere of a winter wheat (*Triticum aestivum* L.) in Denmark by employing bacterial colony based infrared imaging and MALDI-TOF mass spectrometry (MS) techniques. A total of ∼4,480 colonies were screened for the presence of cellular BChl *a*, resulting in 129 AAP isolates that were further clustered into 21 groups based on MALDI-TOF MS profiling, representatives of which were sequenced using the Illumina NextSeq and Oxford Nanopore MinION platforms. Seventeen draft and four complete genomes of AAPs were assembled belonging in *Methylobacterium*, *Rhizobium*, *Roseomonas*, and a novel *Alsobacter*. We observed a diverging pattern in the evolutionary rates of photosynthesis genes among the highly homogenous AAP strains of *Methylobacterium* (Alphaproteobacteria), highlighting an ongoing genomic innovation at the gene cluster level.

## Introduction

Plant–microbe interactions both above (phyllosphere) and below (rhizosphere) ground are common in nature. Traditionally, these relationships are investigated in the rhizosphere, where conditions are relatively stable and nutrient availability is rather high (Vorholt 2012; Carvalho and Castillo 2018). Despite recent work (reviewed in e.g., Lindow and Brandl 2003; Vorholt 2012), the microbe–phyllosphere (which is comprised by the aerial parts—and especially the leaves—of plants) interactions remain relatively understudied compared with rhizosphere. Models suggest that the total leaf surface is >1 billion km^2^ (Woodward and Lomas 2004), potentially colonized by up to 10^26^ bacterial cells (Lindow and Brandl 2003).

Although the phyllosphere seems to be a common environment for bacteria to thrive in, it may be “extreme” for a plethora of reasons. First, different plants exhibit different growth patterns and climate adaptations. Annual plants complete their life cycle in just one growth season, whereas perennial plants grow and shed leaves every year. This creates a discontinuous, ever-changing habitat (Vorholt 2012). Meanwhile, environmental changes also affect the phyllosphere and its inhabitants. Winds, rainfall, frost, and drought all play important roles in shaping the conditions encountered by microorganisms of the phyllosphere. However, the most significant environmental driver is sunlight. Temperature differences in the upper leaves, especially those that form the canopy of various habitats may range up to 50 °C between day and night. Sunlight, though beneficial for photosynthetic organisms, is also comprised of UV-radiation, which is damaging to the DNA of both prokaryotes and eukaryotes (Remus-Emsermann et al. 2014). In addition to the abiotic factors that make the phyllosphere a much more hostile environment than the rhizosphere, its inhabitants also face—directly or indirectly—the influence of their plant host. Leaves are surrounded by a thin, laminar, and waxy layer, the cuticle, which renders the leaf surface hydrophobic, thus removing the excess of water that is collected due to rainfall, dew, or respiration from the stomata. This leads to the retention of little water most usually in veins and other cavities of the cuticle. These microformations also protect the colonizers from the surrounding environment (Beatie 2002). Apart from niche competition and scarce water availability, phyllosphere colonizers also need to compete for nutrients, as the cuticle makes the surface virtually impermeable to nutrients deriving from diffusion through the cells of the plant host (Wilson and Lindow 1994a, 1994b). At the same time, they also need to protect themselves from potential invaders and a plethora of antimicrobial compounds of prokaryotic or eukaryotic origin (Vorholt 2012).

Albeit the harsh conditions they encounter, phyllosphere microogransims exhibit remarkable biodiversity, which, in certain cases, can be comparable to that of human gut microbiome (Xu and Gordon 2003; Knief et al. 2012). Through recent genomics and metagenomics studies, several bacterial genera, such as *Methylobacterium*, have been shown to be ubiquitous in the phyllosphere (Knief et al. 2010) and their role in nutrient recycling, plant growth promotion and protection has been evidenced previously (Beattie and Lindow 1999; Gourion et al. 2006; Atamna-Ismaeel and Finkel 2012a; Kwak et al. 2014). One of the most interesting, recent findings in phyllosphere microbiota was the presence of aerobic anoxygenic phototrophic (AAP) bacteria and rhodopsin-harboring bacteria in a variety of land plants (Atamna-Ismaeel and Finkel 2012a, 2012b). AAP bacteria are commonly found in aquatic environments ranging from the arctic to the tropics and from high salinity lakes to pristine high altitude lakes (Yurkov and Hughes 2017). They rely on organic carbon compounds to cover their nutritional requirements and can also utilize light to produce energy in the form of ATP, without fixing carbon or producing oxygen. AAPs have been found in a variety of extreme environments and have shown their potential to outgrow nonAAP bacteria under nutrient limiting conditions (Hauruseu and Koblížek 2012; Ferrera et al. 2017). Thus, their presence in the phyllosphere may not be surprising. However, their relative abundance, at least in the rice phyllosphere (*Oryza sativa*), was up to three times higher than what is commonly found in marine ecosystems (Atamna-Ismaeel and Finkel 2012a).

These early culture-independent metagenomics studies provided valuable information about the presence of AAP bacteria in the phyllosphere. However, no pure cultures of AAPs have been described so far from phyllosphere and thus detailed genome information is lacking, which prevents an in-depth understanding of the ecological roles and evolutionary trajectory of AAPs in this unique niche. To expand our genomics views of this ecologically important group of bacteria in the phyllosphere, we designed this study with the following aims: 1) to create the first collection of AAP isolates from the phyllosphere, 2) to characterize their photosynthesis gene clusters (PGCs) and compare their gene contents and molecular evolutionary patterns, and 3) to expand the database of complete genomes of AAP bacteria from nonaquatic environments. By combining colony infrared (IR) imaging, MALDI-TOF mass spectrometry (MS) and Illumina and Oxford Nanopore sequencing technologies, we were able to provide 20 draft genomes that contain complete PGCs and four complete genomes that contain plasmids. The further comparison of the evolutionary trajectories of their PGCs revealed a diverging pattern among the highly homogenous *Methylobacterium* strains, highlighting an ongoing genomic innovation at the gene cluster level.

## Materials and Methods

### Sample Collection

A whole intact plant of winter wheat (*Triticum aestivum* L.) with a height of circa 60 cm was collected from a field in Roskilde, Denmark, on June 6, 2018 and transported to the lab for bacterial isolation on the same day. Wheat leaves were cut off and cleaned with running sterile water to remove dust and other temporary deposits from the ambient environment. Microbiota were collected from eight pieces of leaves by rinsing the leaves in 80 ml PBS solution (pH 7.4) in a Ø 150-mm petri dish and scraping the leave surface repeatedly with sterile swabs until the PBS solution slightly turned greenish due to the fall-off of leave cells. From the supernatant, 14 ml were plated on 1/5 strength R2A plates (resulting in a total of 137 agar plates) and incubated at room temperature with normal indoor light conditions for two weeks. AAP bacteria were identified using a custom screening chamber equipped with an IR imaging system, described in detail previously (Zeng et al. 2014). In short, plates were placed in a chamber sealed from light and exposed to a source of green lights. AAP bacteria containing bacteriochlorophyll absorb the green light and emit radiation in the near IR spectrum, which was captured by a NIR sensitive CMOS camera. The image was processed and the glowing colonies indicated the presence of BChl *a*. The colonies that give positive signals were marked, restreaked onto 1/5 strength R2A plates and incubated at room temperature for 72 h. Verification of the light absorbing capabilities of the isolated strains was performed using the same setup. Strains that passed the verification step were further restreaked onto 1/5 strength R2A plates to ensure pure colonies of single AAP strains.

### Selection of AAP Strains and Whole Genome Sequencing

The isolated AAP strains were grouped and dereplicated using a MALDI-TOF MS (Microflex LT, Bruker Daltonics, Bremen, Germany) before performing whole genome sequencing to reduce the cost while maintaining the biodiversity. Briefly, a toothpick was used to transfer a small amount of an AAP bacterial colony onto the target plate (MSP 96 polished steel, Bruker) that was evenly spread out and formed a thin layer of biomass on the steel plate. The sample was then overlaid with 70% formic acid and allowed for air dry before addition of 1 μL MALDI-MS matrix solution (α-cyano-4-hydroxycinnamic acid, Sigma–Aldrich). A bacterial standard with well-characterized peaks (Bruker Daltonics) was used to calibrate the instrument. The standard method “MBT_AutoX” was applied to obtain proteome profiles within the mass range of 2–20 kDa using the flexControl software (Bruker). The flexAnalysis software (Bruker) was used to smooth the data, subtract baseline and generate main spectra (MSP), followed by a hierarchical clustering analysis with the MALDI Biotyper Compass Explorer software, which produced a dendrogram as the output for visual inspection of similarities between samples. An empirical distance value of 50 was used as the cutoff for defining different groups on the dendrogram, corresponding to different strains/species.

From each group on the dendrogram, one isolate was chosen and restreaked on ½ R2A plates and incubated at room temperature for one week. Prior to DNA isolation, the plates were again tested for the presence of light absorbing pigments as previously described. Bacterial colonies were scraped from the plates using a loop and immersed in 400 µL PBS solution, vortexed until the cells had separated, spun down in a table-top centrifuge at 10,000 × g at 4 °C, the supernatant was removed and the pellets were redissolved in milliQ H_2_O. High molecular weight DNA was extracted from each strain using a modified Masterpure Complete DNA & RNA Purification Kit (Lucigen) by replacing the elution buffer with a 10 mM Tris–HCl—50 mM NaCl (pH 7.5–8.0) solution at the final step. DNA concentration was quantified on a Qubit 2.0 (Life Technologies) using the Broad Range DNA Assay kit and DNA quality was measured on a Nanodrop 2000C (Thermo Scientific). Whole genome shotgun sequencing was performed on all AAP strains on the Illumina Nextseq platform in house using the 2×150 bp chemistry. Selected strains were also sequenced on the MinION platform (Oxford Nanopore Technologies, UK) in house using the Rapid Barcoding Kit (SBQ-RBK004) and the FLO-MIN106 flow cell (R9.4.1) following the manufacturer’s instructional manuals.

### Genome Assembly and Analyses

The Illumina pair end reads were trimmed for quality and ambiguities using Cutadapt (Martin 2011) and assembled using SPAdes (Bankevich et al. 2012). The resulting assemblies were analyzed using dRep (Olm et al. 2017) to compare their average nucleotide identities (ANI) in order to identify clusters of closely related taxa. The assemblies were imported in Geneious R.11.2.5 (Biomatters Ltd.) and PGCs were annotated using the *Roseobacter litoralis* strain Och149 plasmid pRL0149 (CP002624) as reference under relaxed similarity criteria (40%). The suggested genes of probable PGCs were analyzed using BLAST (megablast, BlastN) to verify their annotations. For full genome annotations, the assemblies were uploaded on RAST (Aziz et al. 2008; Overbeek et al. 2014; Brettin et al. 2015). The predicted, translated protein coding genes were imported in CMG-Biotools (Vesth et al. 2013) for pairwise proteome comparisons using the native blastmatrix program.

Hybrid assemblies of pair-end Illumina reads and long Oxford Nanopore Technologies reads were performed using the Unicycler assembler (Wick et al. 2017) utilizing the bold assembly mode. The resulting complete genomes were imported in Geneious, where their circularity was confirmed by mapping-to-reference runs using the short and long reads from the respective hybrid assemblies and the native Geneious mapper under default settings in the Medium–Low sensitivity mode.

### Phylogenetic Analyses

Identification of the 16S ribosomal DNA sequences was performed by rnammer in CMG-Biotools. Data sets containing the different genes from the identified PGCs were created in Geneious. The resulting sequences were aligned using MAFFT v.7.388 (Katoh et al. 2002) and it FFT-NS-I x1000 algorithm with default settings as implemented in Geneious. Phylogenetic trees were created using RAxML (Stamatakis 2006) employing the GTR-GAMMA nucleotide substitution model and the rapid bootstrapping and search for best scoring ML-tree with 100 bootstraps replicates and starting from a random tree. Analysis of the PGCs was performed on five selected genes that were present in all isolates, namely *acsF, bchL, bchY, crtB, pufL*, and *pufM.* Individual gene alignments and phylogenetic trees were performed as described above. A super-matrix (8.240 characters) comprised of the total seven gene alignments was created using the built-in “Concatenate alignments” function in Geneious, and a phylogenetic tree was created as above. All trees were visually inspected for disagreements. Finally, a phylogenomic tree was constructed in RAxML employing the GAMMA BLOSUM62 substitution model and the rapid bootstrapping and search for best scoring ML-tree algorithm with 100 bootstraps replicates and starting from a random tree, using as input the core protein alignment consisting of 6.988 characters created with CheckM (Parks et al. 2015) and its lineage_wf algorithm with default settings. Substitution rates were calculated as distances from the root, using the legend provided by the phylogenetic trees.

## Results

### Identification and Isolation of AAP Bacteria from the Phyllosphere

The 129 phyllosphere isolates that were tested positive for the presence of bacteriochlorophyll-α were placed in 21 groups according to their protein mass profiles based on MALDI-TOF MS (supplementary fig. MALDI-TOF, Supplementary Material online). A representative from each group was sequenced (Illumina) and their genomes assembled (SPAdes). WL1 and WL2 were from the same MALDI-TOF group and their genomes are identical (ANI, 100%), confirming the validity of MALDI-TOF MS for grouping similar isolates. Information about their phylogeny, total genome size, and genome assembly statistics are presented in table 1.

**Table 1:** Genome Statistics and Phylogenetic Information of the 21 Isolates that were Illumina Sequenced in This Study

Strain	Taxonomy	# Contigs	Genome Size	N50	GC %
WL1	*Methylobacterium*	654	6,258,106	19,384	68.92
WL2	*Methylobacterium*	298	6,292,310	52,057	68.99
WL3	*Rhizobium*	55	5,333,961	210,875	61.14
WL4	*Alsobacter*	139	5,412,272	86,934	67.92
WL6	*Methylobacterium*	708	5,549,874	15,034	69.72
WL7	*Methylobacterium*	431	6,059,272	36,478	69.04
WL8	*Methylobacterium*	225	5,322,599	56,100	69.69
WL9	*Methylobacterium*	221	4,670,253	42,885	67.41
WL12	*Methylobacterium*	271	5,545,758	53,038	69.4
WL18	*Methylobacterium*	1,373	5,796,709	7,880	68.64
WL19	*Methylobacterium*	330	5,177,712	31,633	66.98
WL30	*Methylobacterium*	377	5,636,306	38,077	69.78
WL45	*Roseomonas*	2,056	5,961,800	5,511	69.54
WL64	*Methylobacterium*	365	6,908,402	48,840	68.34
WL69	*Methylobacterium*	225	4,693,485	43,639	69.51
WL93	*Methylobacterium*	719	5,642,324	16,902	69.45
WL103	*Methylobacterium*	1,327	5,167,285	7,154	68.97
WL116	*Methylobacterium*	1,173	5,445,855	8,413	69.43
WL119	*Methylobacterium*	263	5,709,790	45,485	69.58
WL120	*Methylobacterium*	395	5,266,148	26,698	69.54
WL122	*Methylobacterium*	2,024	4,508,072	3,666	68.77

**Fig. 1.**
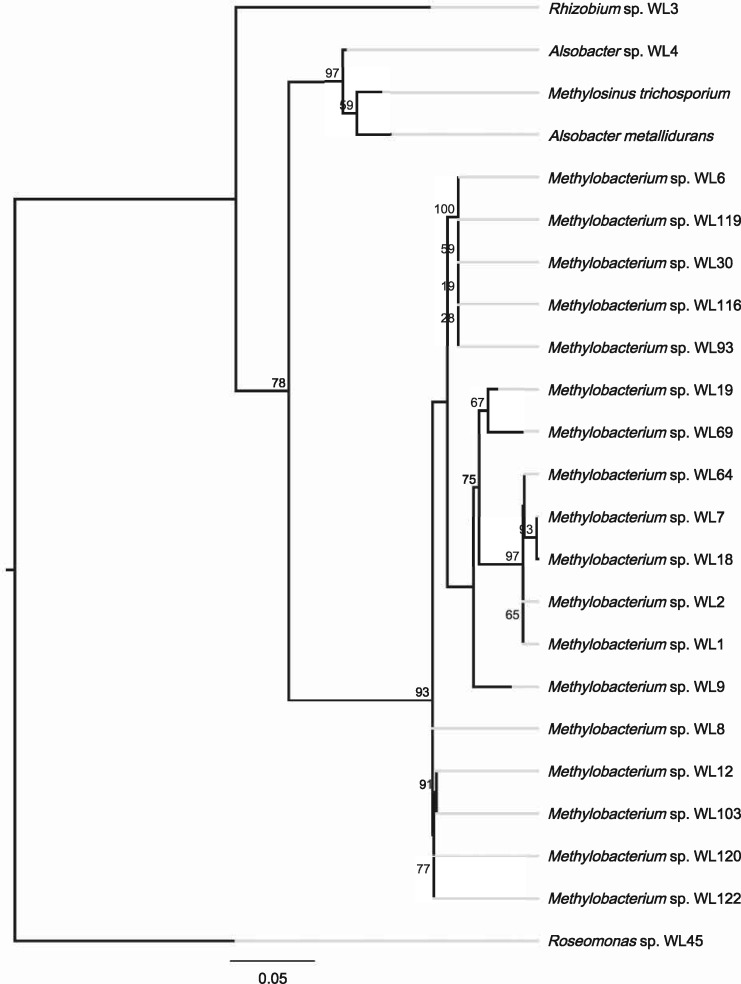
—Phylogenetic tree of the 16S genes of the sequenced isolates. *Methylosinus trichosporium* and *Alsobacter metallidurans* were included to better pinpoint the phylogenetic placement of strain WL4. The legend shows substitution rates from the root of the tree and the node labels indicate bootstrap support values (%).

Analysis of the 16S genes showed that 17 isolates belonged in *Methylobacterium* (separated in four distinct groups), whereas the remaining three belonged to *Rhizobium, Roseomonas* and one novel species was affiliated to the Methylocystaceae family (WL4), with most hits belonging to uncultured, environmental strains. The only characterized hits of WL4 belonged to *Methylosinus trichosporium* (Gorlach et al. 1994) and *Alsobacter metallidurans*, a recently characterized novel genus, novel species (Bao et al. 2006). However, in both instances, the similarity was low (∼98.5%). We included strains of both species and created a 16S phylogenetic tree (fig. 1), which placed *Alsobacter* sp. WL4 as sister to the *Alsobacter-Methylosinus* clade.

**Fig. 2.**
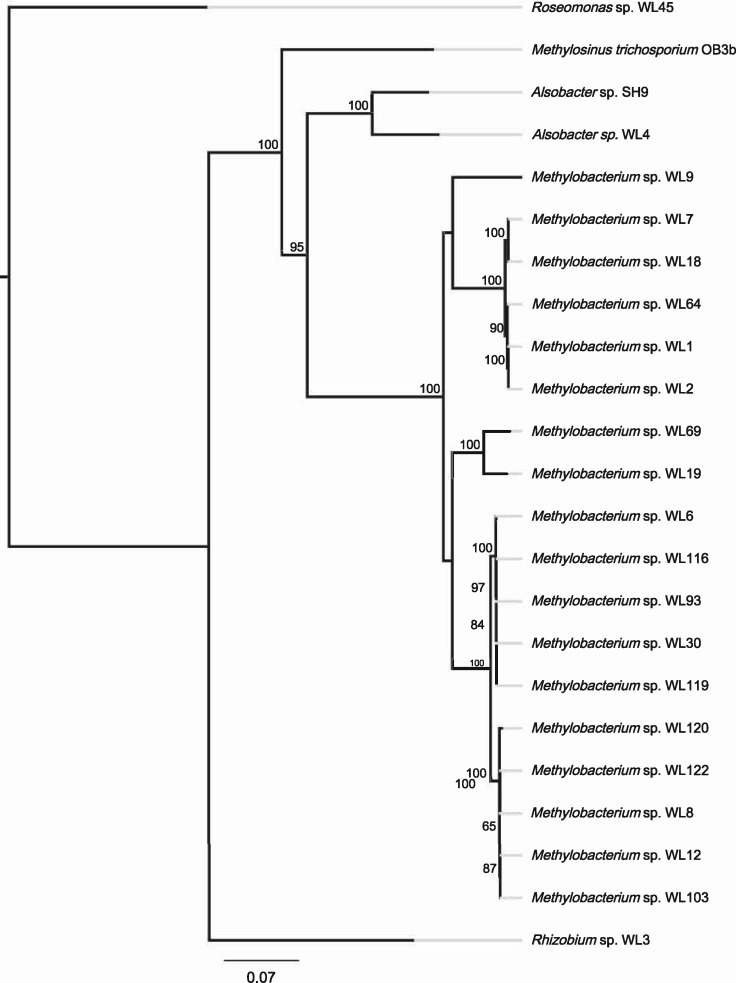
—Phylogenomic tree based on the core proteome (CheckM) of the 21 pure bacterial isolates of the analysis, including *Methylosinus trichosporium* OB3B and *Alsobacter* sp. SH9. The legend shows substitution rates from the root of the tree and the node labels indicated bootstrap support values (%).

**Fig. 3.**
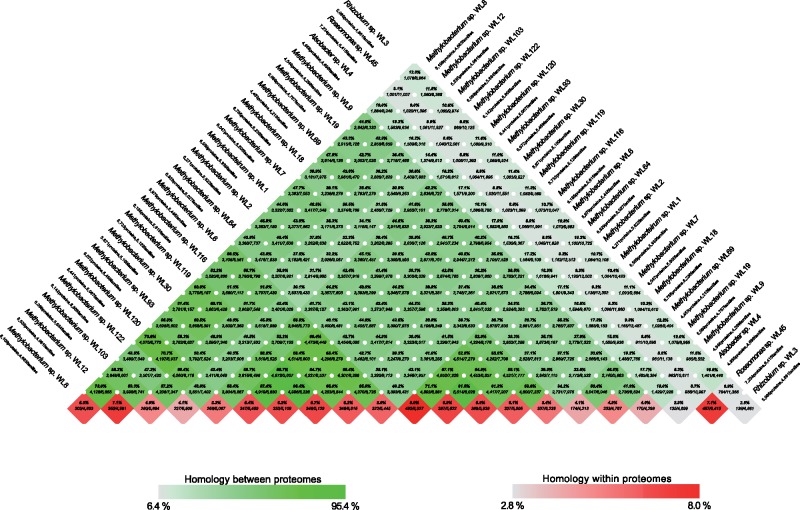
—Pairwise proteome comparisons (BLAST-matrix) of the 21 bacterial isolates of the analysis. The gray-to-green color gradient indicates low to high percentages of identity. The bottom boxes show the presence of homologs within the same proteome (gray-to-red color gradient shows low to high presence of homologs in the specific proteome).

The core proteome analysis (fig. 2—CheckM) including *M. trichosporium* OB3b and *Alsobacter* sp. SH9 established the phylogeny of strain WL4 and it was highly supported as sister to *Alsobacter* sp. SH9 (BS = 100%), both of which were distinct from *M. trichosporium* OB3b (BS = 100%). *Methylobacterium* strains were still placed into four distinct groups (Group 1: WL9, WL19, WL69; Group 2: WL1, WL2, WL7, WL18, WL64; Group 3: WL6, WL30, WL93, WL116, WL119; Group 4: WL8, WL12, WL103, WL120). This was further supported by the whole proteome comparison (fig. 3—BLAST matrix). For Groups 3 and 4, the average core protein content is ∼4.500 proteins, whereas for Group 2 it is close to 4.000. This may signify the presence of either more or larger plasmids that characterize the strains of this group. For Group 1, this number falls even more as in this case there clearly are three distinct species that make up the group.

**Fig. 4.**
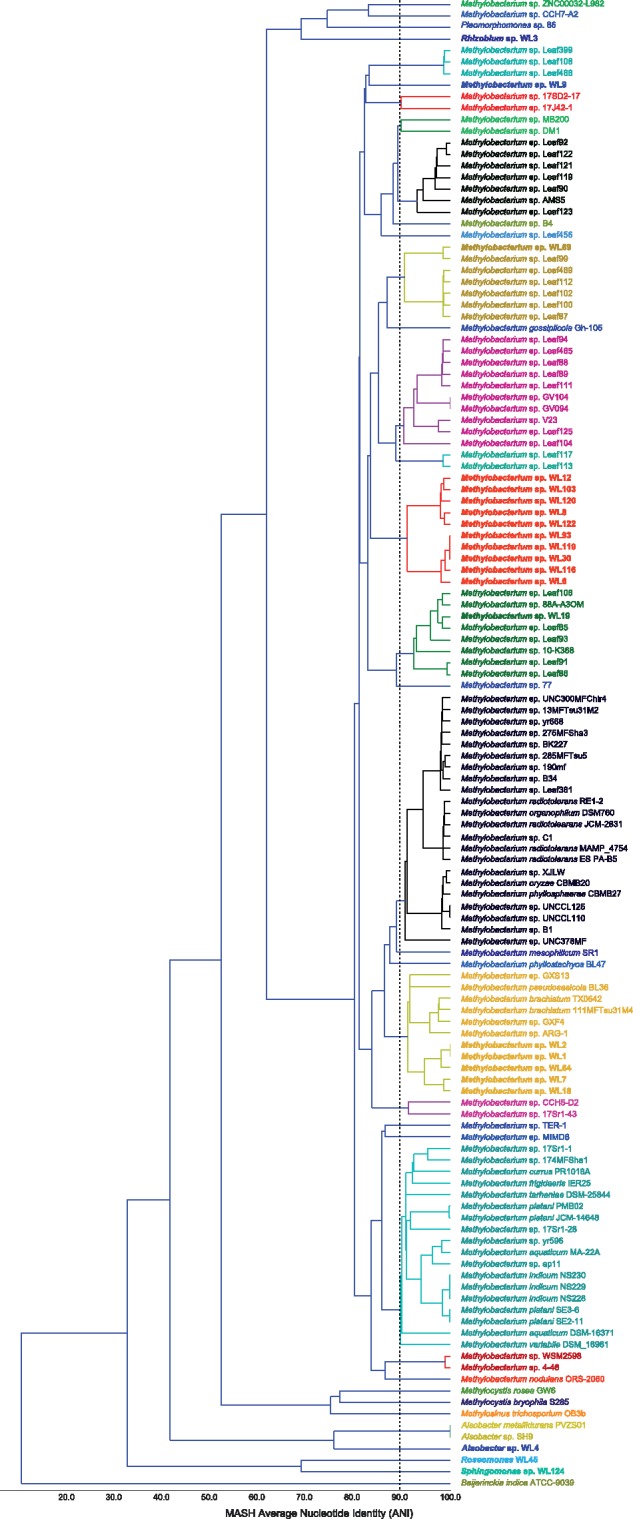
—Whole genome, average nucleotide identity (ANI) cladogram showing percentages of k-mer identities between the isolates. Taxa with the same color and above 90% identity threshold are considered to belong in the same species. *Beijerinckia indica* ATCC-9039 was used as outgroup of the analyses (not defined a priori).

We expanded the analysis to include 100 complete and draft *Methylobacterium* spp. genomes available in GenBank (last assessed April 2019), and also other representatives from the Methylocystaceae, aiming at further establishing strain WL4 in the genus *Alsobacter* and also providing greater resolution on the *Methylobacterium* isolates, with a special focus on the divergent group 1. Information about the included genomes is given in supplementary table 1, Supplementary Material online. We performed whole genome comparisons using ANI (fig. 4—dRep).

The analysis showed that Group 1 isolates belonged in different *Methylobacterium* species, groups 3 and 4 were different strains of the same species (MASH ANI identity between groups > 90%), whereas group 3 isolates are closely related strains, possibly belonging to *Methylobacterium brachiatum*. Strain WL4 is again placed close to the two identical *Alsobacter* genomes (*Alsobacter* sp. SH9 and *A. metallidurans* PVZS01), far away from the three remaining species from within the Methylocystaceae (*M.trichosporium* OB3b, *Methylocystis rosea* GW6, *Methylocystis bryophila* S285). However, the MASH-ANI identity between WL4 and *Alsobacter* is below the 80% threshold of genus assignment. Therefore, we took the top 200 megablast hits for WL4’s 16S region (information provided in supplementary table 2, Supplementary Material online), aligned them including strain WL4 as mentioned previously and created a phylogenetic tree of the 201 sequences (supplementary fig. 1, Supplementary Material online). The results clearly show that WL4 belongs in *Alsobacter*, whereas *Methylosinus* spp. form their own distinct cluster.

### Molecular Evolution of the PGC

The PGCs identified in the sequenced isolates all belong to RC-II type, meaning they contain bacteriochlorophyll-α and possess pheophytin—quinone type reaction centers. All strains possess a full bacteriochlorophyll synthase gene set, except for *Alsobacter* sp. WL4 that is lacking the *bchM* gene (table 2).

**Table 2:** Gene Content of the Photosynthesis Gene Clusters Identified in the 21 Bacterial Isolates

			Bacteriochlorophyll Synthase Genes												Carotenoid Biosynthesis Genes								Light-Harvesting Complex Genes						
	Strain	*acsF*	*bchB*	*bchC*	*bchF*	*bchG*	*bchI*	*bchL*	*bchM*	*bchN*	*bchP*	*bchX*	*bchY*	*bchZ*	*crtA*	*crtB*	*crtC*	*crtD*	*crtE*	*crtF*	*crtI*	*crtK*	*pucC*	*pufA*	*pufB*	*pufC*	*pufL*	*pufM*	*puhA*
	*Rhizobium* sp. WL3																												
	*Roseomonas* sp. WL45																												
	*Alsobacter* sp. WL4																												
Group1	*Methylobacterium* sp. WL9																												
	*Methylobacterium sp.* WL19																												
	*Methylobacterium* sp. WL69																												
Group 2	*Methylobacterium* sp. WL1																												
	*Methylobacterium* sp. WL2																												
	*Methylobacterium* sp. WL7																												
	*Methylobacterium* sp. WL18																												
	*Methylobacterium* sp. WL64																												
Group 3	*Methylobacterium* sp. WL6																												
	*Methylobacterium* sp. WL30																												
	*Methylobacterium* sp. WL93																												
	*Methylobacterium* sp. WL116																												
	*Methylobacterium* sp. WL119																												
Group 4	*Methylobacterium* sp. WL8																												
	*Methylobacterium* sp. WL12																												
	*Methylobacterium* sp. WL103																												
	*Methylobacterium* sp. WL120																												
	*Methylobacterium* sp. WL122																												

Note.—Gray boxes indicate presence of genes and white boxes indicate absence of genes.

For the carotenoid biosynthesis genes, all strains possess *crtB*, whereas *crtA* is only present in *Alsobacter* sp. WL4 and *Rhizobium* sp. strain WL3. For the *crtC* and *crtK* genes an interesting pattern is observed: *Methylobacterium* group 1 and group 2 strains contain *crtC* (with three exceptions, discussed below), whereas group 3 and 4 strains completely lack the gene. On the other hand, *crtK* is only present in *Methylobacterium* group 3 strains and missing from groups 1, 2, and 4. Similar patterns are evidenced in *pufB* which is absent from *Methylobacterium* groups 3 and 4, *Rhizobium* sp. WL3 and *Roseomonas* sp. WL45, and *pufC* missing from *Methylobacterium* group 4 as well as the novel strain WL4. The remaining genes were found ubiquitously in all strains of the analysis, with only a few exceptions (table 2).

**Fig. 5.**
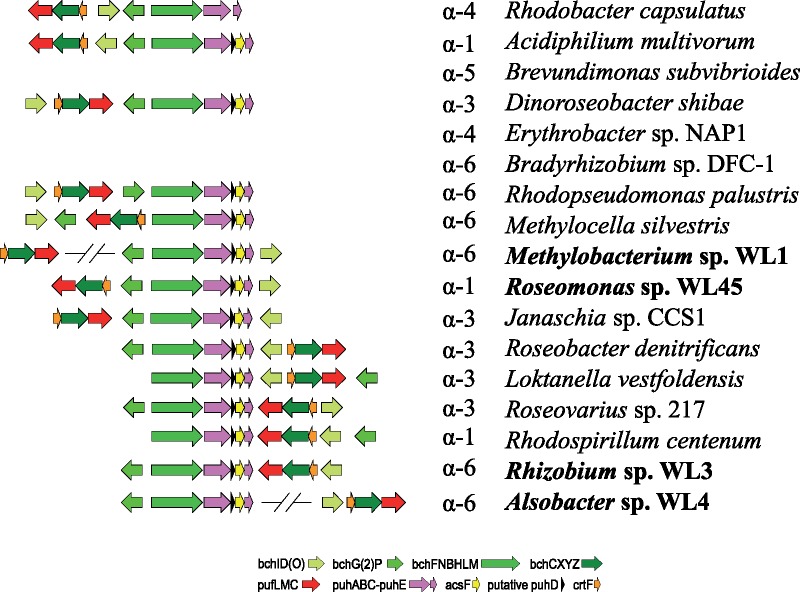
—Architecture of the different photosynthetic gene clusters. Arrows indicate the orientation of the genes. Sizes are not representatives of gene length. Only the genes shared by all PGCs were shown. Genes that may be missing from some strains are denoted with parentheses on the figure’s legend.

The PGCs were consistently found on the longer contigs of the initial assemblies of Illumina data produced by SPAdes (for the strains with fewer than 500 contigs) (table 1). In *Rhizobium* sp. WL3 and *Roseomonas* sp. WL45 the photosynthetic genes form one continuous cluster, with different, however, architectures. In *Alsobacter* sp. WL4 the genes are organized in two clusters, similar to the *Methylobacterium* spp. isolates. In these cases, the genes were found in the middle of the contigs, ruling out the possibility of PGC being organized into two clusters as an artifact of the assembly. Unique to the architecture present in WL4 is the presence of *bchI* before the *crtB, crtI* genes in the first cluster. For *Methylobacterium* spp., groups 2 and 3 show similar gene synteny—though there are a few differences in gene content (table 2)—whereas groups 1 and 4 appear to also share the overall architecture of the PGCs. For isolates WL9 and WL19 (both in group 1) the exact location of the *crtB* and *crtI* genes is not known as they appear on a short contig by themselves. Annotating the assemblies on RAST revealed several housekeeping genes present in the vicinity of these clusters, which further supported the notion of their chromosomal placement. The completed genomes resulting from the hybrid assemblies (strains WL1, WL3, WL4, and WL45) provided the final evidence of the exact placement of the PGCs on the chromosomes of all our isolates. The different architectures of the isolated PGCs are shown in figure 5. General descriptive statistics of the assembled complete genomes are given in table 3. The hybrid assemblies resulted in both complete genomes and plasmids. Thus, *Rhizobium* sp. WL3 contains two plasmids, *Roseomonas* sp. WL45 contains two chromosomes and seven plasmids; *Alsobacter* sp. WL4 with two plasmids; *Methylobacterium* sp. WL1 with one plasmid.

**Table 3:** General Descriptive Statistics of the Four Complete AAP Bacterial Genomes

Taxon	Genome Size (bp)	GC%	Number of Genes	Plasmids	Plasmids Size (bp)	GC%
*Rhizobium* sp. WL3	4,568,855	61.60	4,450	1	700,631	58.6
* *	—	—	—	2	85,350	57.5
*Roseomonas* sp. WL45	4,533,887	70.8	5,042	1	262,815	65.5
* *	1,011,854	70.7	1,138	2	240,556	66.2
* *	—	—	—	3	152,922	65.8
* *	—	—	—	4	85,279	64.6
* *	—	—	—	5	84,774	66.1
* *	—	—	—	6	83,273	65.6
* *	—	—	—	7	65,860	64.8
*Alsobacter* sp. WL4	5,405,668	67.9	5,013	1	27,178	62.0
	—	—	—	2	9,451	60.6
*Methylobacterium* sp. WL1	6,215,463	69.1	6,431	1	35,731	63.8

Using *Roseomonas* sp. WL45 as outgroup we generated phylogenetic trees for the 16S, *acsF, bchL, bchY, crtB, pufL*, and *pufM* genes, as described previously (data not shown). We calculated substitution rates from the root of the different trees, including the core proteome (CheckM) tree and compared them to each other (table 4).

**Table 4:** Substitutions Per Site in the Different Phylogenetic Analyses

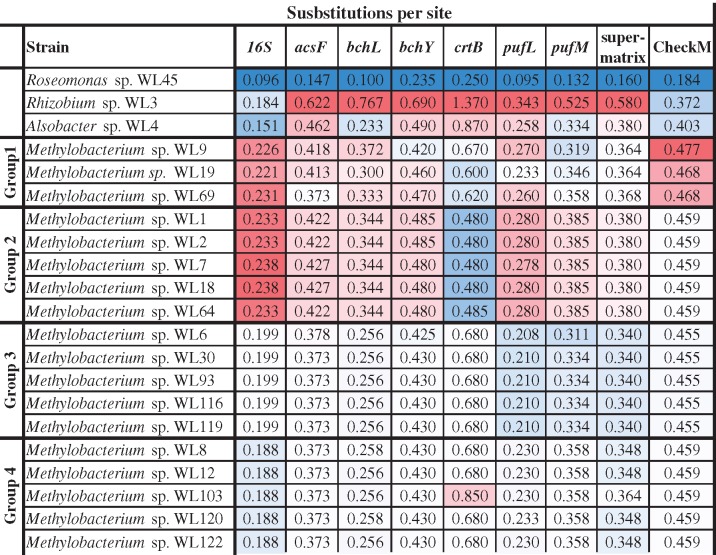

Note.—All values correspond to distances from the root, where *Roseomonas* sp. WL45 was chosen as outgroup for all the alignments.

## Discussion

### A New Strategy to Generate Complete Genomes of Unique Aerobic Anoxygenic Phototrophic Bacteria From Environmental Isolates

Aiming to enhance our knowledge of the prevalence of aerobic anoxygenic phototrophic bacteria in the phyllosphere, we designed this study using wheat as the plant of choice, and employed a high-throughput, multi-disciplinary approach to identify, isolate and analyze AAP strains. From the initial 137 plates, we ended up with ∼4,480 bacterial colonies, >500 of which were probable AAP strains. After macroscopic investigation of size, shape, color, and texture of the colonies, we chose 129 strains, for which we ran proteome profiling using MALDI-TOF MS. This approach has been shown to be able to reproducibly distinguish different taxa, and in cases where reference MALDI-TOF spectra are available in the database, be able to assign Genus and/or Species names (Madsen et al. 2015). We then proceeded with whole genome sequencing on the Illumina Nextseq platform, and for the unique genomes we also proceeded with Oxford Nanopore Technologies sequencing to generate complete genomes and plasmids.

Following this multi-disciplinary approach that relies on classical microbiological techniques (plating, morphology, etc.), biochemical analyses (MALDI-TOF MS), and two different types of high-throughput whole genome sequencing, we were able to generate draft and complete genomes of novel AAP bacteria from the wheat phyllosphere in a relatively quick (weeks) and efficient manner, without losing novel biological information in the process of reducing redundancy.

### Bacteriochlorophyll-Containing AAP Bacteria in the Wheat Phyllosphere

By investigating the presence of bacteriochlorophylls in the wheat phyllosphere, we were able to identify 21 unique strains harboring them, the majority of which belonged to the genus *Methylobacterium*. Members of this genus are ubiquitous in nature and have been detected in both soil and plant surfaces (Green 2006). Targeting the *bchY* (chlorophyllide reductase subunit Y) and *pufM* (reaction centre subunit M) genes in phyllosphere samples of different plants, it was shown that the Methylobacteriaceae (alpha-Proteobacteria) comprised 28% of the total *pufM* sequences from the amplicon sequencing analysis (Atamna-Ismaeel and Finkel 2012a). Our results are, thus, in accordance to these previous studies. As Methylobacteriaceae are indigenous inhabitants of the phyllosphere and have consistently been shown to harbor PGCs, it may be that these photosystems are essential to the Methylobacteria that inhabit this niche. Interestingly, in their study, Atamna-Ismaeel et al. had negative results from their *bchY* amplicon sequencing approach, suggesting the absence of bacteria that contain RC1 (bacteriochlorophyll-α containing reaction centers) type photosystems. Our results show that all isolates contain the complete cassette of chlorophyllide synthase subunit encoding genes (*bchB, bchC, bchF, bchG, bchI, bchL, bchM, bchN, bchP, bchX, bchY, bchZ*), as well as both the *pufL* and *pufM* genes (table 2). Testing the same primers (Yutin et al. 2009) in silico against our *bchY* sequences, we had positive results for all of them (data not shown).

Patterns of loss of the carotenoid genes have been observed in other alpha and gamma proteobacteria, like in Rhodobacterales and Sphingomonadales (Zheng et al. 2011). The absence of *crtA* results in the inability to perform the final step in the spheroidene pathway where hydroxyspheroidene is converted to hydroxyspheroidenone. Moreover, the absence of *crtC* in *Methylobacterium* spp. of groups 3 and 4 completely affects both the spheroidene and the spirilloxanthin pathways as its product is essential for the first step of both metabolic pathways starting from neurosporene and lycopene, respectively. This means that strains of these two groups rely on the zeaxanthin pathway to produce carotenoid pigments, and most likely nostoxanthin and erythroxanthin, which, however, do not participate in the light-harvesting process, but rather have a photoprotection role (Noguchi et al. 1992; Yurkov and Csotonyi 2009).

### Phylogenetic Observations

In our study, we sequenced and analyzed 21 aerobic anoxygenic photosynthetic bacteria, most of which belonged to the genus *Methylobacterium*, the presence of which in the environment has already been discussed previously. *Methylobacterium* spp. are facultative methylotrophs and can utilize a variety of C1, C2, C3, and C4 compounds. Such compounds are often found in the phyllosphere due to plant metabolism (Vorholt 2012; Remus-Emsermann et al. 2014). *Methylobacterium* spp. show a wide range in chromosome size and number of plasmids they contain (Marx et al. 2012). The genus is represented well in GenBank with 100 complete or draft genomes (last accessed April 2019). However, most of the sequences do not have species affiliation (fig. 4). Thus, we were not able to assign any of the 18 isolates to specific *Methylobacterium* species, apart perhaps from group 2 (WL1, WL2, WL7, WL18, WL64), which is placed as sister to *M. brachiatum* in the MASH-ANI analysis. Even in this case, though, *M. pseudosasicola* BL36 is also placed in the same clade as *M. brachiatum*, which raises the question of proper nomenclature. This is further evidenced in the same tree (cyanide clade below) where seven species share >90% of their genome. For the remaining isolates, WL19 is closely affiliated to other *Methylobacterium* strains that have not been identified. A similar picture is shown for WL69. On the other hand, WL19 appears to be a novel species with unique genomic features, and so do the strains from groups 3 and 4 (red clade). Thus, despite how ubiquitous *Methylobacterium* is in nature, it is clear that part of its divergence is still missing. Moreover, a critical evaluation of the current nomenclature of the genus would be beneficial based on the whole genome sequences, because closely related genomes (MASH-ANI identity > 97%) have been given different species affiliations.

Apart from this genus, we also identified a *Rhizobium* sp., a *Roseomonas* sp., and a novel *Alsobacter* strain (WL4) that belongs in the Methylocystaceae, a family of methanotroph bacteria. This is the first time that AAP bacteria from this family have been isolated and their complete genomes assembled. Its closely related *Alsobacter* spp. have been shown to exhibit high Thallium tolerance (Bao et al. 2006), and it will be interesting to identify heavy-metal resistance genes in nonheavy-metal polluted phyllosphere samples. It is worth mentioning that if WL4 contains heavy-metal resistance genes, on top of the PGC, this will have been shown for the first time and its potential to bioremediate heavy-metal contaminated areas if applied as plant growth promotion and environmental remediation agent is of high value and needs to further be explored.

### Molecular Evolution of the PGCs

For all gene trees of the PGC we observed that *Rhizobium* sp. WL3 evolves faster than the other isolates, followed by *Alsobacter* sp. WL4. Both these isolates also show much higher substitution rates compared with their respective 16S genes and core proteomes (checkM), which indicates that the PGCs are under relaxed selection pressure. In all instances, the phylogenetic trees constructed showed the same topology and the same grouping of the *Methylobacterium* isolates, which suggests that the PGCs were incorporated in the genomes of these strains relatively early in their evolution. If the PGC was free to move laterally then we would have observed very different phylogenetic trees for its genes compared with the 16S and core proteome analyses. Interestingly, these observations do not reflect on the architecture of the PGC, where *Roseomonas* sp. WL45 and *Rhizobium* sp. WL3 contain the PGC in one continuous region, whereas *Alsobacter* sp. WL4 and *Methylobacterium* sp. WL1 have it in two pieces.

For the 18 *Methylobacterium* isolates, group 1 and 2 strains (WL9, WL19, WL69 and WL1, WL2, WL7, WL18, WL64) evolve consistently at different rates compared with the groups 3 and 4. It appears that there is relatively strong selection pressure on *bchL*, *pufL*, and *pufM*, which in all cases are more similar to each other and also show slower molecular evolution rates compared with *ascF, bchY*, and *crtB*, which in all *Methylobacterium* isolates evolve at least twice as fast compared with the 16S gene (table 4). They also show fewer substitutions per site compared with the core proteome analysis. This result partially contrasted with previously adopted methods of using *bchY* as a marker gene for RC1 clusters (e.g., Atamna-Ismaeel and Finkel 2012a). On the other hand, the use of *pufM* appears more sensible. It would, however, be more suitable to investigate the possibility of using other genes for metagenome amplicon sequencing approaches, such as *pufL* or *bchL*, which show a smaller degree of divergence in different genera, thus universal primers might be more successful in detecting a wide variety of AAP bacteria in environmental samples. Interestingly, while Groups 1 and 2 isolates exhibit higher substitution rates of the PGC genes compared with Groups 3 and 4, this is not the case for *crtB* which evolves a lot slower, and also *pufM*, which shows similar molecular evolution in the genus. Overall, it seems that the PGCs were incorporated in ancestral strains that later diverged, to give rise to the different AAP taxa. This is further corroborated by the fact that almost all phylogenetic trees (e.g., fig. 5) are consistent and in agreement with both the 16S phylogenetic tree (fig. 1) as well as the phylogenomic tree (fig. 2). If the genes of the PGCs had been transferred to these AAP several times, there would have been significant differences both in the sequence level and the phylogenetic analysis of the different genes, which would be expected to result in different topologies and diverse distances from the root, even for closely—based on 16S analysis—related strains.

### The Significance of AAP Bacteria in Wheat Phyllosphere

In this study, we isolated for the first time a variety of AAP bacteria that are present in the wheat phyllosphere, adding to the metagenomics knowledge provided earlier from five other plants (Atamna-Ismaeel and Finkel 2012a, 2012b). The phyllosphere is a rather harsh environment, with little water availability, scarce resources, very few suitable locations, extreme temperature difference between day and night, and high dosages of UV radiation (Vorholt 2012). AAP bacteria have the ability to utilize light in photophosphorylation processes, through which they produce ATP and at the same time funnel the few available nutrients in other metabolic pathways (Yurkov and Hughes 2017). This has been shown to give them an edge in vitro after exposure to light, compared with nonAAP bacteria (Ferrera et al. 2011). Considering that phyllosphere bacteria protect the plant from pathogenic microbes by secreting metabolites and by occupying the available niches, being able to persevere and outgrow other bacteria gives a significant edge to AAP taxa and probably points toward the suggestion of positive selection of such bacteria in their phyllosphere by the plants themselves, as they appear to be absent from the soil (Atamna-Ismaeel and Finkel 2012b). At this point, it is not clear exactly how AAP bacteria benefit the plant, especially since the phyllosphere is a complex habitat with an ever-changing, harsh environment. In the past years, great efforts have been carried out on how to improve crop production by studying the rhizosphere. It has also been suggested that the phyllosphere communities play a significant role in plant health and growth, which ultimately leads to increased yields (Lindow and Brandl 2003). Given the current projections of world population growth and the supply of food (UN 2017), it becomes evident that more, in-depth studies investigating the phyllosphere of plants, and especially crops, and identifying the underlying drivers of the bacterial communities and their interactions with their plant hosts is of paramount importance. Thus, by isolating, maintaining and analyzing the genomes of phyllosphere isolates, we may identify strains that may prove useful as plant growth promoting agents in future field trials investigating the effect of phyllosphere inoculation on plant growth, health, and yield.

## Supplementary Material

Supplementary data are available at *Genome Biology and Evolution* online.

## Supplementary Material

evz204_Supplementary_Data

## Notes

**Data deposition:** This project has been deposited at GenBank under the accession numbers: SAMN12513103–SAMN12513123.
