# Supplementary figures and images for: Genomics of Aerobic Photoheterotrophs in Wheat Phyllosphere Reveals Divergent Evolutionary Patterns of Photosynthetic Genes in Methylobacterium spp
Source: Genome Biol Evol. 2019 Sep 17;11(10):2895–908. doi: 10.1093/gbe/evz204 (PMC6798729; doi:10.1093/gbe/evz204)

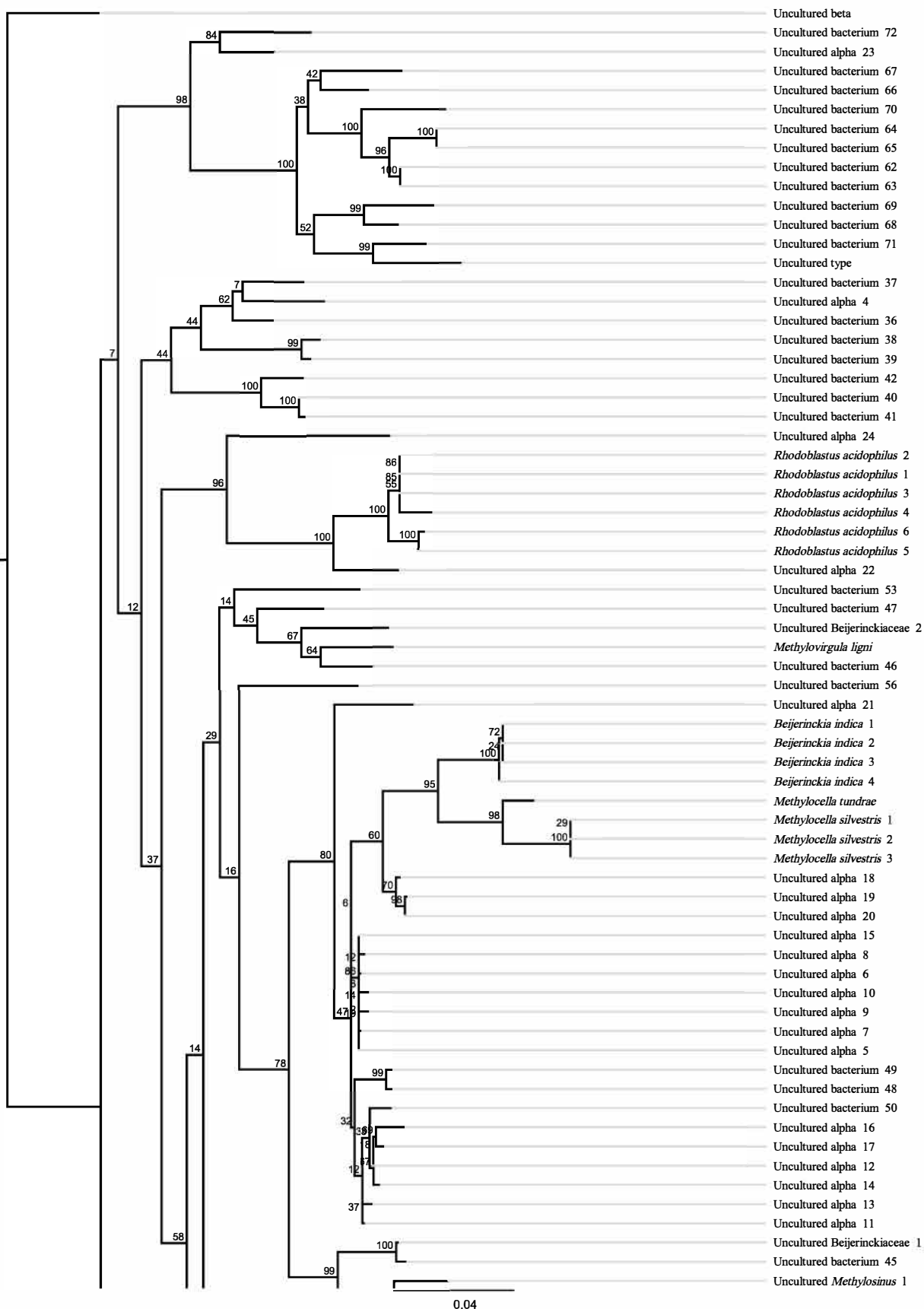

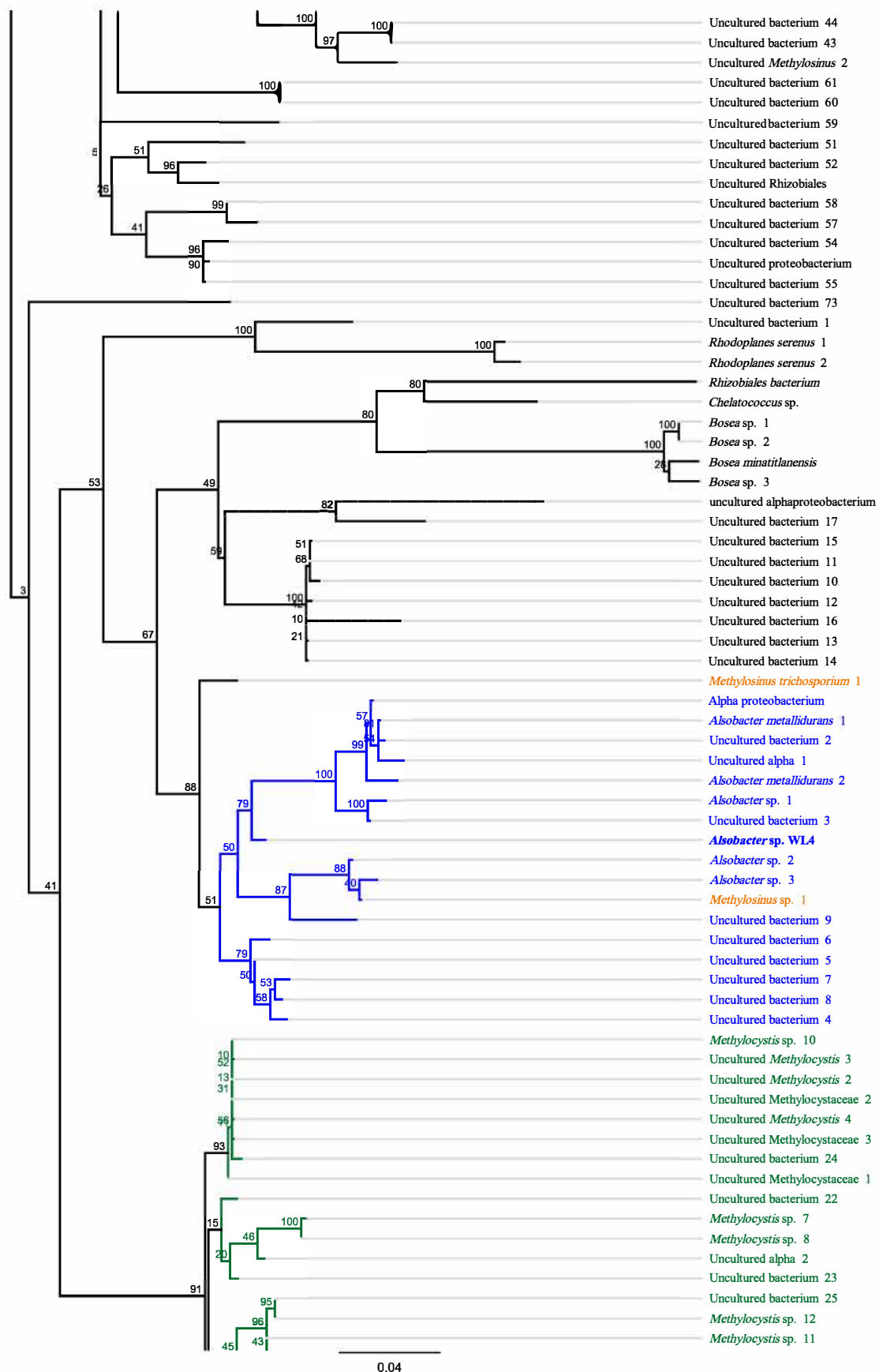

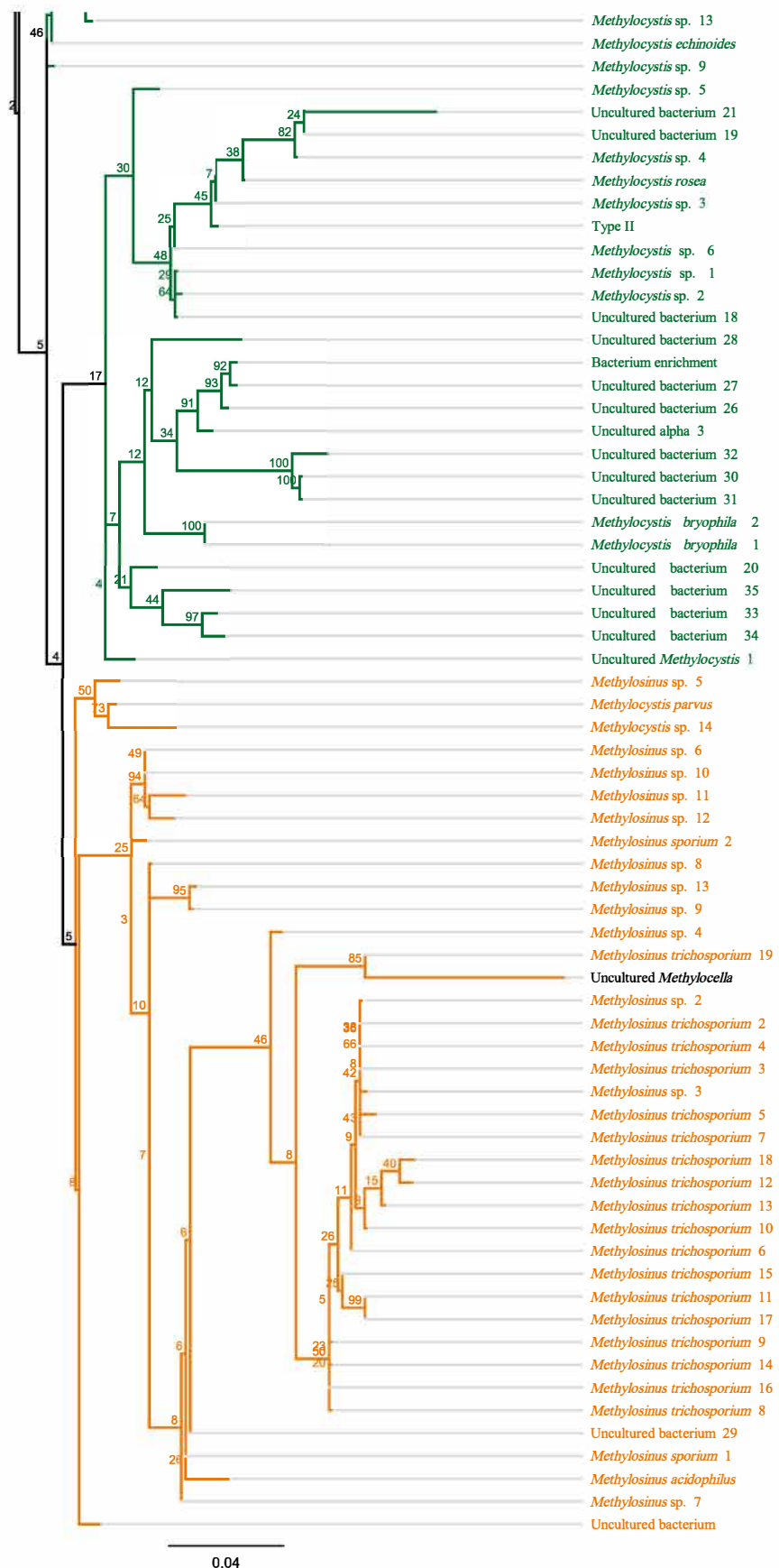

Supplement: evz204_Supplementary_Data [file evz204_supplementary_data.zip › Supp_mat_Fig1_Alsobacter_16S_200NCBIhits_final.pdf]

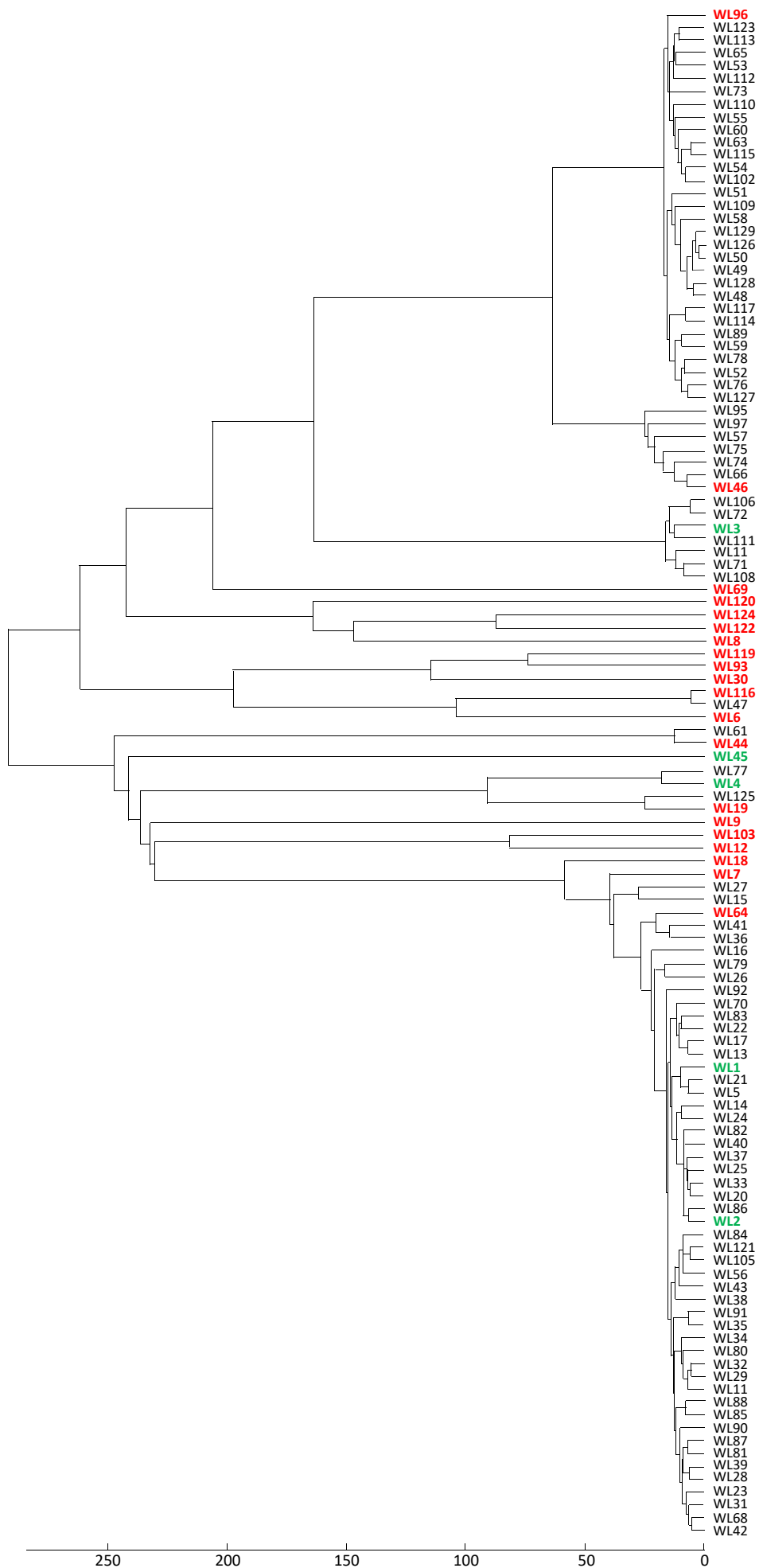

Supplement: evz204_Supplementary_Data [file evz204_supplementary_data.zip › Supp_mat_Fig2_MALDI_TOF.pdf]
